# Ten-year panel data confirm generation gap but climate beliefs increase at similar rates across ages

**DOI:** 10.1038/s41467-021-24245-y

**Published:** 2021-07-06

**Authors:** Taciano L. Milfont, Elena Zubielevitch, Petar Milojev, Chris G. Sibley

**Affiliations:** 1 School of Psychology, University of Waikato, Waikato, New Zealand; 2 School of Psychology, University of Auckland, Auckland, New Zealand

**Keywords:** Climate change, Environmental social sciences, Psychology and behaviour, Psychology

## Abstract

Accumulating evidence indicates that climate change awareness and concern has increased globally, but commentators suggest a climate change generation gap whereby younger people care more about climate change than older people. Here we use a decade of panel data from 56,513 New Zealanders to test whether belief that “Climate change is real” and “Climate change is caused by humans” increased over the 2009-2018 period; and whether changes are uniform across 12 five-year birth cohorts spanning those born from 1936 to 1995. Results confirm a generation gap in mean (intercept) climate change beliefs but not in over-time increase (slope). The generation gap occurs because older cohorts started from a lower initial belief level (circa 2009), but all age cohorts increased their belief level at a similar rate over the last decade; and these results were not qualified by respondents’ gender. The findings offer hope for collective action that bridges efforts across generations.

## Abstract (web summary)

It has been suggested that younger people care more about climate change than older people. Here, the authors present ten year panel data from New Zealand and show that despite a generation gap in starting levels, climate change beliefs have increased at similar rates across ages over the 2009-2018 period.

## Introduction

Opinion polls indicate awareness of and belief in climate change are increasing over time. In 2020 a total of 73% of polled respondents in the USA said global warming is happening, representing a ten-percentage increase since 2015 (ref. ^1^). Another survey confirmed USA respondents have grown increasingly worried about climate change between 2010 and 2019 (ref. ^2^). In New Zealand, the number of polled respondents who feel the issue of climate change is important to them personally grew from 72 to 79% in the 2018–2019 period alone^3^.

This increase in climate change concern and beliefs over time is evidenced in many other countries around the world. According to data from the Pew Research Center the median of respondents in 23 countries who said climate change was a major threat to their country increased from 56% in 2013 to 67% in 2018 (ref. ^4^). Climate change was also rated the top concern in half of these countries when compared to seven other global threats, such as the condition of the global economy and cyberattacks.

Findings indicating that public concern about climate change is increasing over time are coupled with expressed sentiments that current leaders do not care enough about the consequences of climate change because they will not be directly affected by or will not have to solve the issue^5–9^. Commentators have described this sentiment as the climate change generation gap^10–14^, suggesting that young people are more concerned about protecting the environment and addressing climate change than older people. This sentiment was made even more salient after the school strike for climate pioneered in August 2018 by the climate and environmental activist Greta Thunberg^15^, the resulting Global Climate Strike for Future organized in March 2019, and the ongoing Fridays for Future movement.

But is there empirical evidence for the climate change generation gap? Notwithstanding the logical sentiment expressed by young people that climate change is more pressing to them because they will live to experience its personal and societal consequences, existing research provides mixed evidence for a generation gap regarding climate change. On the one hand, some research findings indicate that young people are indeed more concerned about protecting the environment and addressing climate change, and express greater climate distress^16–20^. Data from over 27,000 citizens across the 28 European Union countries also show that those aged 15–24 years old are more likely to be concerned about climate change than those aged 55 and over^21^.

On the other hand, however, this age effect is not observed consistently^2,22–24^. Findings from a meta-analysis of 794 correlations between age and pro-environmental variables across 87,988 unique individuals indicate most variables had no or negligible relationships with age—but older individuals appear more likely to engage with nature and in behaviours aimed to conserve natural resources and avoid environmental harms, and to do so due to societal norms^24^. Moreover, the 2018 data from the Pew Research Center mentioned above showed reliable age differences in only four countries: respondents aged 18–29 years old from Australia, France, Philippines, and the USA were more likely to say that climate change was a major threat to their country than those aged 50 and over, with no reliable age differences in the other 19 countries^4^.

These contrasting results indicate the evidence for a climate change generation gap is mixed and might be dependent on particular environmental questions considered or the particular country investigated. Notably, most of the studies examining age effects rely on cross-sectional data. Though it is informative to document patterns of change at the population level and age associations with climate change questions, research designs typically used in opinion polls do not afford examination of the extent to which climate change beliefs of a particular individual change over time. Such research designs are also limited in examining whether over-time change is observed in all age cohorts of a population.

Here, we confirm a generation gap in mean levels but not in over-time increase for both the statements “Climate change is real” and “Climate change is caused by humans” using a decade of panel data from New Zealand. The generation gap occurs because older cohorts start from a lower initial belief level, but climate beliefs increase at similar rates across ages over the 2009–2018 period and the results are comparable for women and men.

## Results and discussion

Existing evidence suggests an increase in climate change beliefs over time coupled with mixed evidence indicating younger people express greater climate change beliefs than older people. This begs the question of whether over-time change in climate change belief represents a developmental process or a cohort effect. On the one hand, perhaps there is an ageing effect that matches the expressed negative association between age and climate change beliefs such that as people age they believe less in climate change. We consider this unlikely. On the other hand, perhaps people of all ages are increasing their belief in climate change; it is only that older people are starting at a lower level of climate belief. We consider this possibility more plausible as it would account for both observations from existing research indicating an increase in climate change belief over time, coupled with an inverse relationship between climate change belief and age observed in some cross-sectional studies. Of course, this does preclude the possibility that the rate of increase of climate belief may differ in magnitude across age cohorts, such that younger people may be accelerating in their belief compared to older people.

To examine these questions, we analysed data from the New Zealand Attitudes and Values Study (NZAVS), which is a longitudinal national probability study that has been assessing people’s socio-political attitudes annually since 2009. Panel data allow examination of the development of climate change beliefs for the same individual over time, as well as afford better examination of temporal change in climate change beliefs than cross-sectional public opinion polls. Building on recent advances in structural equation models, we use a data analytical approach that disentangles age and cohort effects by examining whether changes in climate change beliefs increase or decrease with the same rate of change across different age cohorts.

### Disentangling over-time change and age effects in climate change beliefs

Across survey waves, respondents expressed their levels of agreement with two statements: “Climate change is real” and “Climate change is caused by humans”. We report data collected across ten waves of annual assessments from 2009 (Time 1) to 2018 (Time 10), and model an accelerated cohort design to estimate ageing and cohort effects in the rate of growth in agreement levels to these climate change beliefs across the adult lifespan.

To assess ageing and cohort effects, we estimated three complementary multi-group cohort-sequential latent growth models^25–27^. The age-based model assumes a normative ageing effect for climate change beliefs from age 18 to 78. The cohort-based model allows for the possibility that changes in climate change beliefs may vary across birth cohorts, and complements the first model by allowing assessment of cohort effects in the estimated change trajectories in climate change beliefs across the adult lifespan. We also estimated an intermediate cohort-based model that assumes birth cohorts vary in their starting point of climate beliefs, but that all birth cohorts’ rate of change in these beliefs are the same over time. This partially constrained model represents an intermediate version between the age-based trajectory model, which has a common intercept and common growth rate, and the cohort-based trajectory model, which allows both intercepts and slopes to vary.

Cohorts and year of birth were computed based on respondents’ age at the time of data collection. We considered 12 five-year birth cohorts based on year of birth that spans seven decades with people born from 1936 to 1995. Sample sizes for the five-year birth cohorts ranged from 495 respondents in the 1940–1936 cohort to 8819 respondents in the 1960–1956 cohort (see Table 1). The “Methods” section provides details about sample characteristics, sampling procedure, and model estimations.

**Table 1:** Age and sample sizes by birth cohort for each climate change belief.

			Sample sizes	
Birth cohorts	Age at Time 1 (~2009)	Age at Time 10 (~2018)	Climate change reality	Climate change caused by humans
1995–1991	18	27	3404	3399
1990–1986	19	28	4297	4295
1985–1981	24	33	4396	4395
1980–1976	29	38	5066	5061
1975–1971	34	43	6092	6087
1970–1966	39	48	7003	6986
1965–1961	44	53	8216	8202
1960–1956	49	58	8819	8799
1955–1951	54	63	6265	6246
1950–1946	59	68	1566	1565
1945–1941	64	73	940	937
1940–1936	69	78	495	495
Total *N*	—	—	56,559	56,467

The youngest age in each birth cohort was taken as indication of age at Time 1.

### Generation gap in starting levels but not in rate of over-time increase in climate change beliefs

**Fig. 1:**
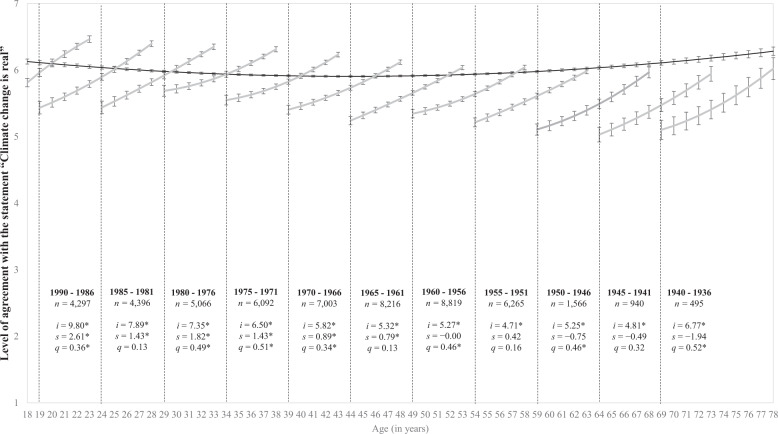
Belief in the reality of climate change across age and five-year birth cohorts. Model-implied change trajectories in the level of agreement with the statement “Climate change is real” (dark line) from ages 18 to 78. The lines within each 5-year cohort represent longitudinal change in climate reality, estimating latent intercepts (*i*) as well as linear (*s*) and quadratic (*q*) slopes. The estimations are based on the mean-levels of climate reality (*y*-axis) across age and assessments (*x*-axis) with 95% confidence intervals presented as error bars around each point estimate (*p < 0.05). Due to graphical space constraints, details for the 1995–1991 cohort are not shown but are: *n* = 3404, *i* = 6.00*, *s* = −1.45, *q* = −0.56*. Respondents expressed their levels of disagreement-agreement on a 7-point answer scale anchored by 1 (strongly disagree) and 7 (strongly agree).

**Fig. 2:**
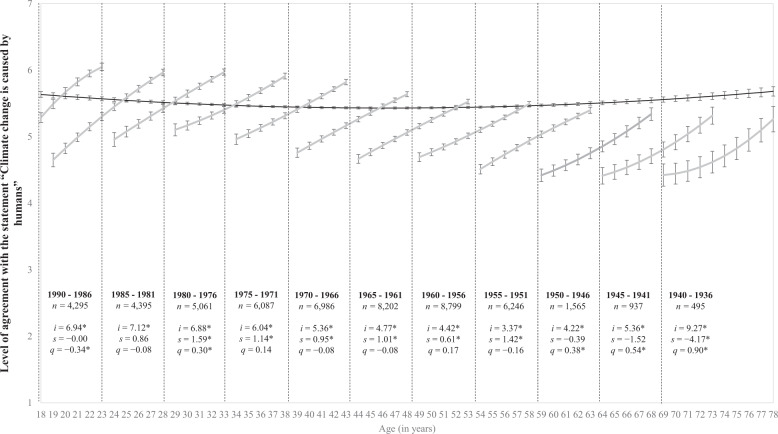
Belief in anthropogenic climate change across age and five-year birth cohorts. Model-implied change trajectories in the level of agreement with the statement “Climate change is caused by humans” (dark line) from ages 18 to 78. The lines within each 5-year cohort represent longitudinal change in human causation belief, estimating latent intercepts (*i*) as well as linear (*s*) and quadratic (*q*) slopes. The estimations are based on the mean-levels of human causation belief (*y*-axis) across age and assessments (*x*-axis) with 95% confidence intervals presented as error bars around each point estimate (*p < 0.05). Due to graphical space constraints, details for the 1995–1991 cohort are not shown but are *n* = 3399, *i* = 1.72, *s* = −4.82*, *q* = −1.29*. Respondents expressed their levels of disagreement–agreement on a 7-point answer scale anchored by 1 (strongly disagree) and 7 (strongly agree).

Respondents expressed their levels of disagreement–agreement to the climate change beliefs on a 7-point answer scale anchored by 1 (strongly disagree) and 7 (strongly agree). Results from the age-based model indicate that agreement with the reality of climate change was higher at all age groups than agreement with anthropogenic climate change. The mean agreement level for climate change reality at the sample mean age (about 46 years of age; or 46.17 (SD = 14.03) specifically) was 5.91 (95% CI [5.89, 5.92]), and the mean agreement level for anthropogenic climate change was 5.43 (95% CI [5.42, 5.45]). This pattern of higher agreement levels for the reality of climate change than for anthropogenic climate change is evident when contrasting the darker lines in Figs. 1 and 2.

Beyond this distinction in overall mean levels, mean agreement levels regarding both the reality of climate change and its human cause followed a non-linear trajectory across the adult lifespan. Results in Table 2 confirm that the longitudinal changes in climate change beliefs did not follow a linear increase but a quadratic change trajectory for both climate change beliefs, as indicated by the statistically significant quadratic slopes (*p* values < .001). The darker lines in Figs. 1 and 2 visually depict a U-shaped pattern in agreement levels from ages 18 to 78. Respondents’ belief in both the reality of climate change and belief in anthropogenic climate change was comparatively higher in early adulthood, lower in middle adulthood, and higher again in older ages. Visual inspection of these figures indicates the shift from lower-to-higher agreement levels started at a younger age for belief in human causation (around age 45) when compared to belief in climate reality (around age 50).

**Table 2:** Parameter coefficients for the cohort-constrained models for climate change reality and human causation estimating the change trajectory from ages 18 to 78.

Belief	Estimate	SE	Est./SE	*P* value	95% CI lower	95% CI upper	Variances
Climate change reality							
Intercept	5.91	0.01	836.34	<0.001	5.89	5.92	1.19*
Linear slope	0.01	0.00	1.40	0.162	−0.00	0.01	0.07*
Quadratic slope	0.03	0.00	12.06	<0.001	0.03	0.04	0.00
Climate change caused by humans							
Intercept	5.43	0.01	683.91	<0.001	5.42	5.45	1.61*
Linear slope	−0.01	0.01	−1.46	0.144	−0.02	0.00	0.13*
Quadratic slope	0.03	0.00	8.16	<0.001	0.02	0.03	0.00

*p < 0.001.

Results in Table 3 indicate that forcing a normative ageing effect to the data was less fitting than a cohort-based trajectory model allowing variability in each of the 12 five-year birth cohorts. Chi-square results in Table 4 comparing the two models clearly indicate that the age-based trajectory model had relatively worse fit (reflected by higher chi-square values) for all birth cohorts compared to the cohort-based (unconstrained) model. These findings indicate variability in the pattern of longitudinal changes across age cohorts.

**Table 3:** Fit statistics for the age-based and cohort-based trajectory models for each climate change belief.

		Fit statistics												
Belief	Model	*χ*^2^	df	*P* value	Δ*χ*^2^	Δdf	CFI	ΔCFI	RMSEA	ΔRMSEA	SRMR	ΔSRMR	AIC	Sample-size adjusted BIC
Climate change reality	Age-based trajectory (constrained)	19,744.90	773	<0.001	*—*	*—*	0.749	*—*	0.072	*—*	0.175	*—*	481,353.93	481,394.29
	Cohort-based trajectory (constrained slopes)	11,911.51	762	<0.001	7833.39	11	0.853	−0.104	0.056	0.016	0.126	0.049	473,542.54	473,646.31
	Cohort-based trajectory (unconstrained)	11,707.13	740	<0.001	204.38	22	0.855	−0.002	0.056	0.000	0.131	−0.005	473,382.16	473,612.77
Climate change caused by humans	Age-based trajectory (constrained)	17,275.69	773	<0.001	*—*	*—*	0.808	*—*	0.067	*—*	0.126	*—*	502,352.96	502,393.31
	Cohort-based trajectory (constrained slopes)	8284.12	762	<0.001	8991.57	11	0.913	−0.105	0.046	0.021	0.078	0.048	493,383.39	493,487.13
	Cohort-based trajectory (unconstrained)	8095.93	740	<0.001	188.19	22	0.914	−0.001	0.046	0.000	0.081	−0.003	493,239.20	493,469.73

*χ*^2^ chi-square, df degrees of freedom, CFI Comparative Fit Index, RMSEA root mean square error of approximation, SRMR standardized root mean square residual, AIC Akaike Information Criterion, BIC Bayesian Information Criterion.

**Table 4:** Chi-square (*χ*^2^) contributions and differences of each birth cohort for each climate change belief.

	Climate change reality					Climate change caused by humans				
Birth cohort	*χ*^2^A	*χ*^2^B	*χ*^2^ΔAB	*χ*^2^C	*χ*^2^ΔBC	*χ*^2^A	*χ*^2^B	*χ*^2^ΔAB	*χ*^2^C	*χ*^2^ΔBC
1995–1991	1768	1078	689	1032	46	1320	547	773	482	65
1990–1986	2032	1161	872	1139	21	1797	791	1006	764	27
1985–1981	1784	1027	757	1021	6	1472	675	797	674	1
1980–1976	1674	1003	671	970	33	1412	654	758	634	20
1975–1971	1677	986	690	961	26	1617	782	835	772	10
1970–1966	1724	1037	687	1019	18	1631	828	803	822	6
1965–1961	2028	1222	806	1220	1	1707	843	864	838	5
1960–1956	1889	1187	702	1163	24	1639	776	864	769	7
1955–1951	1601	986	615	985	1	1566	759	806	759	0
1950–1946	1563	898	665	885	13	1412	647	765	631	16
1945–1941	1079	625	454	617	8	954	467	487	450	16
1940–1936	926	702	224	695	6	751	517	233	503	14

Chi-square (*χ*^2^) contribution differences (*χ*^2^Δ) between the key competing models are shown. *χ*^2^A Chi-square contribution of the age-based (constrained) model, *χ*^2^B Chi-square contribution of the intermediate model where intercepts are freely estimated but slopes are constrained, *χ*^2^C Chi-square contribution of the cohort-based (unconstrained) model.

We then examined the multi-group cohort sequential models estimating mean-level change over the ten annual assessments in each of the 12 five-year birth cohorts. Results indicate that agreement levels about the reality of climate change and human causation are increasing for people across all age cohorts at about the same rate, but older people are starting from a lower level of belief. As depicted in Fig. 1, respondents’ belief in the reality of climate change had longitudinal patterns of increase for most age cohorts; that is, longitudinal changes were observed in 10 out of the 12 five-year birth cohorts, or in 83.3% of the age cohorts. The exceptions were the 1955–1951 and 1945–1941 cohorts which showed longitudinal stability in agreement levels. Longitudinal changes for climate reality tend to follow a quadratic pattern for the majority of the age cohorts; that is, in 8 out of the 10 five-year birth cohorts where longitudinal change was observed, or in 80% of the cohorts. This indicates that levels of agreement with the statement “Climate change is real” had accelerated increase within each cohort. Given the visual similarities in change trajectories, we also fitted a model that formally tested whether change trajectories for each birth cohort were equal over time, while allowing their intercepts to vary. Results in Table 3 indicate that this intermediate model was commensurate with the main cohort-based model, suggesting that beliefs in the reality of climate change had a similar growth rate for all birth cohorts over 10 years.

Similar patterns of longitudinal changes were observed for belief in anthropogenic climate change as depicted in Fig. 2. Respondents’ belief in human causation had longitudinal patterns of increase in 11 out of the 12 five-year birth cohorts, or in 91.7% of the age cohorts. The exception was the 1985–1981 cohort which showed longitudinal stability in agreement levels. But in contrast to climate reality, levels of agreement with the statement “Climate change is caused by humans” followed a quadratic pattern less frequently; that is, only in 6 out of the 11 five-year birth cohorts where longitudinal change was observed, or in 54.5% of the cohorts. This indicates that levels of agreement regarding human causation had linear longitudinal increases within many age cohorts. Again, we also formally tested the intermediate model in which birth cohorts’ intercepts were freely estimated but their slopes were equivalent across time. Table 3 shows that this model had only trivial differences when compared with the main cohort-based (unconstrained) model. This suggests that although birth cohorts differ in their starting points, their rates of change regarding belief in human causation are similar over time.

Interestingly, for both climate change beliefs the stronger quadratic effect was observed for the 1940–1936 cohort (0.52 and 0.90, respectively). This indicates that the over-time accelerated increase in beliefs regarding the reality and human causation of climate change was more marked for older respondents.

Since there is evidence that pro-environmentalism and climate change concern is greater for women than for men^28–30^, we also extended our test of a generational gap by partitioning our analyses by gender (dummy coded as 0 = women, 1 = men). Results detailed in the Supplementary Information indicates that beliefs in both the reality of climate change and human causation had a similar growth rate for all birth cohorts over 10 years among women and men. This means that the results indicating that birth cohorts differ in climate change beliefs in their starting points but not in their rates of change over time do not depend on (were not moderated by) respondents’ gender.

### Age effects in the age of climate change?

Our findings provide a more nuanced and necessary qualification of the so-called climate change generation gap^10–14^. People born more recently have higher agreement levels about the reality of climate change and its human causation than older people. This observation confirms previous findings drawing from cross-sectional and opinion poll data indicating that young people are more concerned about addressing climate change^16–21^, perhaps because they are the ones who will live to experience more drastic personal and societal consequences^5–9^.

Extending this observation, we were able to examine the extent to which there was also a generation gap in longitudinal changes in climate change beliefs by using data tracking agreement levels in beliefs for the same individuals over time. Our findings document an over-time increase in beliefs about the reality and human causation of climate change over the 2009–2018 period in New Zealand across the life span, in age cohorts with birth spanning seven decades from 1936 to 1995. Although a generation gap was observed at baseline levels of climate change beliefs, there was no generation gap in rate of increase in beliefs, and these findings were not qualified by respondents’ gender.

These findings indicate that, over the last 10 years, all birth cohorts are increasing in their belief that climate change is real and that climate change is caused by humans, suggesting these beliefs are somewhat malleable. Agreement levels in the reality of climate change and its human causation is going up at the same rate but older people are starting from a lower point. That is, a climate change generation gap is present in baseline levels of climate change beliefs, but the rate of increase in levels of climate beliefs does not differ across age cohorts. Both younger and older people are accelerating in their belief at a comparable rate.

It is worth noting that mitigation and adaptation actions to tackle climate change and to adapt to its environmental and societal consequences require breaking from old production, consumption, and institutional habits to innovative ways of doing things, new lifestyles, and envisioning novel collective futures for human societies^31–34^. These necessary changes require openness to new information and ways of doing things, but values and traits related to openness to experience and change decrease as people age^35–38^. These developmental changes in traits related to openness may explain the ageing effects we observed since younger individuals might be more open to engage with the scientific consensus on climate change impacts compared to their older counterparts. However, our findings draw a more nuanced picture by demonstrating that climate change beliefs of older generations are increasing as well.

Moreover, there is an intrinsic temporal delay between environmental problems and their observable and felt consequences, and this means older people will be less affected by the consequences of climate change than younger people. These observations coupled with findings showing that age differences might only emerge for certain measures of people’s environmental appraisal and behavioural responses to environmental issues^24^ suggests age effects in longitudinal changes are still likely for certain issues related to climate change. For example, our results might differ if our survey questions allowed us to examine generational gaps in calls for action against climate change instead of beliefs.

It is worth noting our findings are limited to a single nation and to climate change beliefs measured with single items. Moreover, although the findings were consistent across women and men, there might be other factors (e.g., media awareness of climate change, increased observed negative impacts of climate change) that could explain why older cohorts are starting from lower rates but increasing at similar rates to younger cohorts regarding climate change beliefs.

Future research could build upon our work on a number of ways. First, it is important to examine the extent to which the findings can be replicated and extended beyond the New Zealand population. There are other publicly available longitudinal studies, such as the Understanding Society in the UK^39,40^, that afford examination of the climate change generation gap in other cultural contexts and with other survey measures. Based on our findings, it is likely researchers will observe a generation gap in mean levels of climate-related measures but comparable over-time increase across age cohorts.

Another interesting avenue for future research is examining the extent to which climate communication should be tailored to distinct age groups within the population. Although age is related to certain psychological roots linked to acceptance of climate science (e.g., political ideology)^41^, it is possible age might have an independent influence on the acceptance of scientific communication related to climate change given its impact on climate change beliefs^42^. Experimental studies examining the effectiveness of communication messages to foster climate action for distinct age cohorts would be valuable. Previous research has also investigated drives of pro-environmental activism in young people and how family dynamics influence pro-environmental tendencies^43–46^. It would be interesting to examine such processes regarding climate change beliefs; for example, whether children are more influential in sparking climate action in their parents, whether parents are the catalyst of climate action in their children, or whether the influence process is bi-directional.

A generation gap can be a major barrier for addressing climate change as individuals in positions of power to lead actions tend to be older. The observation that belief in the reality and human causation of climate change has increased over time across all age groups of the New Zealand population—it is the starting point that differs—is thus promising. The findings suggest widespread communication highlighting the seriousness of climate change could be working, and the findings are particularly promising because the seriousness of climate change to human societies require actions from people of all ages and backgrounds.

## Methods

### Panel data

We used ten annual waves of the New Zealand Attitudes and Values Study (NZAVS) to examine ageing versus cohort effects in agreement levels of climate change beliefs. The NZAVS is an ongoing panel study and data are hosted at the University of Auckland, New Zealand. The NZAVS is reviewed every 3 years by the University of Auckland Human Participants Ethics Committee. The most recent ethics approval was renewed on 05 September 2017 until 03 June 2021 (Reference Number: 014889).

The two belief items analysed (i.e., “Climate change is real” and “Climate change is caused by humans”) were embedded in a large battery of Likert-type questions. Respondents expressed their levels of disagreement–agreement to the climate change beliefs on a 7-point answer scale anchored by 1 (strongly disagree) and 7 (strongly agree). Table 1 provides information about the sample considered in our analyses, and full information about the sampling procedure and sample details for each wave is available on the NZAVS website: www.nzavs.auckland.ac.nz.

### Modelling approach

We employed three complementary multi-group cohort-sequential latent growth models^25–27^ to disentangle age-based and cohort-based effects in the two key climate change beliefs. All analyses were computed in Mplus, and the syntax used to test all models reported in this article are available on the NZAVS website: www.nzavs.auckland.ac.nz.

The age-based trajectory model assumes a developmental trajectory over adulthood (i.e., a normative ageing effect). We modelled this by allowing only one overall intercept and rate of growth over 10 years that best fit all birth cohorts simultaneously. We also modelled the rate of change as a polynomial growth function which included linear and quadratic components to allow for the possibility of a curvilinear growth trajectory. Finally, we constrained the variances of the latent intercept, latent growth trajectory—as well as their covariances—to equality across birth cohorts.

In contrast, the cohort-based trajectory model assumes the possibility that each birth cohort differed—specifically, that the time period a person was born in had unique effects on the trajectory of beliefs about climate reality and human causation (i.e., a cohort effect). To model this, we estimated a different latent intercept and latent growth trajectory for each of the 12 five-year birth cohorts. The variances and covariances were still constrained across the birth cohorts. Therefore, any potential differences in fit between the cohort-constrained and unconstrained models represent the extent to which the rate of change over time for individual birth cohorts (i.e., cohort effect) diverged from the rate of change that best fit all cohorts simultaneously (i.e., ageing effect).

Finally, we also modelled an intermediate model between the age-based and cohort-based models, in which the intercepts for each birth cohort were freely estimated, but in which their linear and quadratic slopes were constrained to equality. This allowed us to formally test whether birth cohorts had a similar rate of change over the 10 years, despite having different starting points. The variances and covariances were still constrained across the birth cohorts. However, for visual representation purposes we contrast the age-based and unconstrained cohort-based models in Figs. 1 and 2. The Supplementary Information presents additional results for these models, as well as detailed information about examination of potential moderation of the climate change generation gap by gender.

The youngest age (in years) within a cohort was used as an indicator of age in our models. For example, the 1990–1986 birth cohort represented change from ages 19 to 28 years, and so on. However, there is one exception to this: the 1995–1991 birth cohort spanned ages 18–23 because calculating the values based on the youngest age in this birth cohort at Time 1 (age 14), while theoretically accurate, is not in the observed range because the NZAVS only samples those aged 18 and over. The NZAVS used a force-choice gender question in the first survey waves (i.e., Are you male or female?). The gender question was updated to an open-ended question starting in the Time 6 (2014) survey (i.e., What is your gender?)^47^. Gender was dummy coded (0 = women, 1 = men) in the present models.

As reported in Table 3, the models were contrasted via comparison of relative change in the Comparative Fit Index (CFI), the root mean square error of approximation (RMSEA), and the standardized root mean square residual (SRMR). Given our large sample size, the chi-square test statistic (*χ*²) is unsuitable because it is sensitive to minor differences^48^, but we report it as customary. To examine the fit of each birth cohort, we nevertheless inspected and compared the relative chi-square contribution for each birth cohort (with higher relative values indicating worse fit). This approach allowed us to identify differences that are due to birth cohort effects, and those that are due to normative age-based change over time. Table 4 presents these comparisons in chi-square contributions.

### Reporting summary

Further information on research design is available in the Nature Research Reporting Summary linked to this article.

## Supplementary information

Supplementary Information

Reporting Summary

## Data Availability

Data cannot be made publicly available due to ethical restrictions imposed by the University of Auckland Human Participants Ethics Committee. Full copies of the data files are held by all members of the NZAVS management team and advisory board. A de-identified dataset containing the variables analysed in this article is available upon request from the first author, or any member of the NZAVS advisory board. Contact details for all members of the board are available on the NZAVS website: www.nzavs.auckland.ac.nz. Requests for data access can also be made to the Chair of the University of Auckland Human Participants Ethics Committee, who can be contacted at humanethics@auckland.ac.nz, and more information can be obtained here: https://www.auckland.ac.nz/en/about/research/re-ethics/re-uahpec.html. Such data will be provided with the explicit understanding that they are used solely for the purposes of replicating or checking the validity of analyses/results reported in this and other scientific papers using NZAVS data.
