# Supplementary material for: Ten-year panel data confirm generation gap but climate beliefs increase at similar rates across ages
Source: Nat Commun. 2021 Jul 6;12:4038. doi: 10.1038/s41467-021-24245-y (PMC8260718; doi:10.1038/s41467-021-24245-y)
Supplement: Supplementary file 1 — Supplementary Information [file 41467_2021_24245_MOESM1_ESM.pdf]

Supplementary Information

Article:
Ten-year panel data confirm generation gap but climate beliefs increase at similar rates across ages

Authors:
Taciano L. Milfont[1*], Elena Zubielevitch[2], Petar Milojev[2], and Chris G. Sibley[2]

Affiliations:
[1]School of Psychology, University of Waikato, New Zealand; [2]School of Psychology, University of Auckland, New Zealand

Corresponding author:
Taciano L. Milfont, School of Psychology, University of Waikato, Private Bag 3105, Hamilton 3240, New Zealand. Email: taciano.milfont@waikato.ac.nz

Supplementary Table 1 presents the distribution of responses for the climate change beliefs across ages. This table updates the table provided by Milfont, Wilson and Sibley (2017).

Milfont, T. L., Wilson, M. S., & Sibley, C. G. The public's belief in climate change and its human cause are increasing over time. *PLoS ONE* **12**, e0174246 (2017).

Supplementary Table 1. Distribution of responses to the statements "Climate change is real" (reality) and "Climate change is caused by humans" (cause) in the first ten waves of the New Zealand Attitudes and Values Study, with descriptive statistics for each statement at each wave. Distributions are based on valid percent and sample sizes at each time point refer to those who completed at least one time point of the first ten waves of the New Zealand Attitudes and Values Study.

		Response Option									
		1	2	3	4	5	6	7			
Year	Statement	Strongly Disagree						Strongly Agree	*M*	*SD*	*N*
2009	Reality	4%	4%	6%	14%	15%	24%	34%	5.39	1.66	6062
	Cause	6%	7%	8%	19%	19%	22%	19%	4.82	1.73	6008
2010	Reality	4%	5%	5%	15%	17%	26%	28%	5.29	1.64	4125
	Cause	6%	7%	8%	19%	20%	21%	18%	4.74	1.74	4104
2011	Reality	3%	4%	5%	14%	18%	25%	31%	5.41	1.58	6499
	Cause	6%	7%	8%	19%	20%	23%	18%	4.79	1.72	6455
2012	Reality	3%	3%	5%	13%	16%	26%	34%	5.48	1.59	10079
	Cause	5%	6%	9%	18%	21%	22%	20%	4.88	1.70	10018
2013	Reality	3%	3%	4%	12%	14%	25%	39%	5.60	1.58	17278
	Cause	5%	5%	7%	16%	19%	23%	25%	5.08	1.70	17245
2014	Reality	2%	2%	3%	11%	17%	25%	40%	5.73	1.44	14973
	Cause	3%	4%	6%	16%	21%	23%	27%	5.24	1.60	14925
2015	Reality	2%	2%	3%	9%	15%	26%	43%	5.84	1.41	13283
	Cause	3%	4%	5%	13%	19%	26%	30%	5.36	1.60	13260
2016	Reality	2%	2%	3%	8%	12%	24%	51%	6.02	1.34	20686
	Cause	3%	3%	5%	12%	17%	26%	35%	5.56	1.53	20644
2017	Reality	1%	2%	2%	7%	11%	23%	54%	6.09	1.32	16063
	Cause	3%	3%	4%	11%	16%	26%	37%	5.60	1.53	16012
2018	Reality	2%	2%	2%	6%	9%	19%	59%	6.14	1.37	43473
	Cause	3%	3%	4%	10%	15%	23%	41%	5.64	1.59	43316

Supplementary Table 2. Parameter coefficients for the cohort-based unconstrained models for "Climate change is real" (reality) and "Climate change is caused by humans" (cause)

		Climate Reality						Climate Cause					
						95% CIs						95% CIs	
Birth cohort		Est.	SE	Est./S.E	*p*-value	LB	UB	Est.	SE	Est./S.E	*p*-value	LB	UB
1995-1991	*i*	6.00	1.71	3.52	< .001	2.66	9.35	1.72	1.82	0.94	.346	-1.85	5.28
	s	-1.45	1.37	-1.05	.292	-4.14	1.24	-4.82	1.46	-3.29	.001	-7.69	-1.95
	q	-0.56	0.27	-2.05	.040	-1.10	-0.03	-1.29	0.29	-4.44	< .001	-1.87	-0.72
1990-1986	*i*	9.80	0.69	14.30	< .001	8.46	11.15	6.94	0.73	9.51	< .001	5.51	8.37
	s	2.61	0.67	3.88	< .001	1.29	3.93	-0.00	0.72	-0.01	.996	-1.41	1.40
	q	0.36	0.16	2.21	.027	0.04	0.68	-0.34	0.17	-1.97	.049	-0.68	-0.00
1985-1981	*i*	7.89	0.39	20.48	< .001	7.13	8.64	7.12	0.41	17.42	< .001	6.32	7.92
	s	1.43	0.51	2.82	.005	0.44	2.43	0.86	0.54	1.60	.111	-0.20	1.91
	q	0.13	0.16	0.78	.434	-0.19	0.44	-0.08	0.17	-0.47	.637	-0.42	0.26
1980-1976	*i*	7.35	0.15	48.81	< .001	7.06	7.65	6.88	0.16	43.17	< .001	6.56	7.19
	s	1.82	0.30	6.09	< .001	1.24	2.41	1.59	0.32	5.02	< .001	0.97	2.21
	q	0.49	0.14	3.53	< .001	0.22	0.76	0.30	0.15	2.04	.042	0.01	0.59
1975-1971	*i*	6.50	0.04	184.61	< .001	6.43	6.57	6.04	0.04	160.85	< .001	5.97	6.12
	s	1.43	0.14	10.01	< .001	1.15	1.71	1.14	0.15	7.58	< .001	0.84	1.43
	q	0.51	0.12	4.20	< .001	0.27	0.75	0.14	0.13	1.11	.266	-0.11	0.39
1970-1966	*i*	5.82	0.02	319.28	< .001	5.79	5.86	5.36	0.02	264.13	< .001	5.32	5.40
	s	0.89	0.04	25.06	< .001	0.82	0.96	0.95	0.04	25.18	< .001	0.88	1.03
	q	0.34	0.11	3.07	.002	0.12	0.55	-0.08	0.12	-0.73	.467	-0.31	0.14
1965-1961	*i*	5.32	0.03	214.48	< .001	5.27	5.37	4.77	0.03	177.43	< .001	4.72	4.82
	s	0.79	0.10	8.38	< .001	0.61	0.98	1.01	0.10	10.16	< .001	0.81	1.20
	q	0.13	0.10	1.25	.210	-0.07	0.33	-0.08	0.11	-0.70	.484	-0.29	0.14
1960-1956	*i*	5.27	0.09	60.48	< .001	5.10	5.44	4.42	0.09	48.33	< .001	4.24	4.60
	s	-0.00	0.19	-0.01	.996	-0.38	0.38	0.61	0.20	3.01	.003	0.21	1.00
	q	0.46	0.10	4.47	< .001	0.26	0.66	0.17	0.11	1.55	.122	-0.04	0.38
1955-1951	*i*	4.71	0.22	20.98	< .001	4.27	5.15	3.37	0.24	14.33	< .001	2.91	3.83
	s	0.42	0.33	1.30	.195	-0.22	1.06	1.42	0.34	4.17	< .001	0.75	2.09
	q	0.16	0.11	1.41	.158	-0.06	0.39	-0.16	0.12	-1.35	.176	-0.40	0.07
1950-1946	*i*	5.25	0.49	10.76	< .001	4.29	6.21	4.22	0.51	8.32	< .001	3.23	5.22
	s	-0.75	0.53	-1.42	.156	-1.77	0.28	-0.39	0.55	-0.72	.469	-1.46	0.67
	q	0.46	0.14	3.30	.001	0.19	0.73	0.38	0.14	2.65	.008	0.10	0.67
1945-1941	*i*	4.81	0.99	4.85	< .001	2.86	6.75	5.36	1.03	5.20	< .001	3.34	7.38
	s	-0.49	0.84	-0.58	.562	-2.14	1.16	-1.52	0.88	-1.74	.083	-3.24	0.20
	q	0.32	0.18	1.81	.071	-0.03	0.67	0.54	0.18	2.92	.004	0.18	0.90
1940-1936	*i*	6.77	1.90	3.57	< .001	3.05	10.49	9.27	1.97	4.70	< .001	5.40	13.14
	s	-1.94	1.34	-1.45	.147	-4.57	0.69	-4.17	1.39	-2.99	.003	-6.90	-1.43
	q	0.52	0.24	2.21	.027	0.06	0.98	0.90	0.24	3.66	< .001	0.42	1.37

i = intercept, *s* = linear slope, *q* = quadratic slope.

Supplementary Table 3. Parameter coefficients for the partially-constrained cohort-based models for "Climate change is real" (reality) and "Climate change is caused by humans" (cause)

		Means				95% CIs		Variances	
Climate Reality		Estimate	SE	Est./SE	*p*-value	LB	UB	Est	SE
1995-1991		8.49	0.04	193.57	< .001	8.40	8.57	1.14*	0.01
1990-1986		7.94	0.03	232.61	< .001	7.88	8.01	1.14*	0.01
1985-1981		7.45	0.03	280.98	< .001	7.40	7.50	1.14*	0.01
1980-1976		6.98	0.02	337.76	< .001	6.94	7.02	1.14*	0.01
1975-1971	Intercepts	6.42	0.02	386.42	< .001	6.38	6.45	1.14*	0.01
1970-1966	freely	5.85	0.02	384.76	< .001	5.82	5.88	1.14*	0.01
1965-1961	estimated	5.30	0.02	333.10	< .001	5.27	5.33	1.14*	0.01
1960-1956		4.85	0.02	252.93	< .001	4.81	4.88	1.14*	0.01
1955-1951		4.34	0.03	165.93	< .001	4.29	4.39	1.14*	0.01
1950-1946		3.79	0.04	92.39	< .001	3.71	3.87	1.14*	0.01
1945-1941		3.30	0.06	59.57	< .001	3.19	3.41	1.14*	0.01
1940-1936		2.90	0.08	37.62	< .001	2.75	3.05	1.14*	0.01
All cohorts	Linear slope constrained	0.93	0.01	82.07	< .001	0.90	0.95	0.05*	0.01
All cohorts	Quadratic slope constrained	-0.01	0.00	-1.74	.082	-0.02	0.00	0.00	0.00

		Means				95% CIs		Variances	
Climate Cause		*Estimate*	*SE*	*Est./SE*	*p*-value	LB	UB	Est	SE
1995-1991		8.45	0.05	175.88	< .001	8.36	8.54	1.56*	0.02
1990-1986		7.79	0.04	208.10	< .001	7.71	7.86	1.56*	0.02
1985-1981		7.27	0.03	247.58	< .001	7.21	7.32	1.56*	0.02
1980-1976		6.67	0.02	288.64	< .001	6.62	6.72	1.56*	0.02
1975-1971	Intercepts	6.04	0.02	321.91	< .001	6.00	6.08	1.56*	0.02
1970-1966	freely	5.35	0.02	310.18	< .001	5.32	5.38	1.56*	0.02
1965-1961	estimated	4.73	0.02	264.83	< .001	4.70	4.77	1.56*	0.02
1960-1956		4.20	0.02	198.56	< .001	4.16	4.25	1.56*	0.02
1955-1951		3.64	0.03	126.67	< .001	3.58	3.69	1.56*	0.02
1950-1946		3.02	0.05	65.38	< .001	2.93	3.11	1.56*	0.02
1945-1941		2.53	0.06	40.30	< .001	2.40	2.65	1.56*	0.02
1940-1936		2.03	0.09	23.13	< .001	1.86	2.20	1.56*	0.02
All cohorts	Linear slope constrained	1.04	0.01	86.12	<.001	1.01	1.06	0.10*	0.01
All cohorts	Quadratic slope constrained	-0.03	0.00	-7.39	< .001	-0.04	-0.02	0.00	0.00

* $p < .001$

Supplementary Table 4. Mean and standard deviation of "Climate change is real" for each cohort and survey wave

		Survey Wave (year)									
		1 (2009)	2 (2010)	3 (2011)	4 (2012)	5 (2013)	6 (2014)	7 (2015)	8 (2016)	9 (2017)	10 (2018)
1936-1940	M	5.254	5.060	5.147	5.163	5.367	5.619	5.616	5.688	5.903	5.712
	SD	1.786	1.925	1.749	1.761	1.752	1.447	1.516	1.484	1.400	1.661
	n	319	251	374	368	341	323	294	288	236	233
1941-1945	M	5.123	4.974	5.144	5.216	5.331	5.585	5.592	5.775	5.860	5.899
	SD	1.884	1.825	1.708	1.769	1.723	1.513	1.559	1.518	1.506	1.557
	n	423	340	561	658	637	617	576	587	506	583
1946-1950	M	5.193	5.056	5.208	5.199	5.360	5.511	5.594	5.783	5.875	5.889
	SD	1.745	1.682	1.750	1.769	1.676	1.591	1.566	1.485	1.439	1.606
	n	618	463	735	1048	1093	1066	999	1033	901	994
1951-1955	M	5.297	5.332	5.240	5.329	5.471	5.574	5.698	5.887	5.965	5.969
	SD	1.776	1.641	1.707	1.698	1.705	1.552	1.515	1.480	1.432	1.520
	n	656	494	762	1175	1659	1555	1416	2433	1957	5169
1956-1960	M	5.473	5.363	5.423	5.525	5.537	5.639	5.747	5.929	6.018	6.002
	SD	1.634	1.621	1.566	1.610	1.658	1.507	1.485	1.407	1.407	1.481
	n	727	535	795	1334	2642	2278	2100	3265	2616	7152
1961-1965	M	5.317	5.272	5.339	5.422	5.549	5.662	5.772	5.924	5.992	6.022
	SD	1.654	1.646	1.595	1.611	1.602	1.462	1.441	1.379	1.360	1.445
	n	742	525	776	1287	2489	2219	1966	3064	2352	6560
1966-1970	M	5.496	5.312	5.480	5.581	5.632	5.742	5.846	6.007	6.076	6.108
	SD	1.558	1.625	1.527	1.472	1.561	1.410	1.358	1.335	1.299	1.366
	n	740	481	646	1083	2163	1850	1648	2565	1984	5376
1971-1975	M	5.517	5.418	5.601	5.671	5.690	5.818	5.931	6.082	6.168	6.234
	SD	1.605	1.533	1.417	1.482	1.523	1.396	1.292	1.286	1.248	1.290
	n	582	376	524	894	1842	1565	1350	2184	1661	4695
1976-1980	M	5.666	5.586	5.694	5.853	5.838	5.904	6.072	6.178	6.250	6.326
	SD	1.498	1.391	1.345	1.293	1.412	1.314	1.228	1.177	1.139	1.183
	n	467	266	421	734	1434	1182	1012	1730	1282	3806
1981-1985	M	5.419	5.404	5.730	5.737	5.709	5.956	6.070	6.261	6.333	6.352
	SD	1.600	1.422	1.302	1.354	1.425	1.239	1.186	1.111	1.092	1.184
	n	363	183	315	520	1077	863	727	1326	969	3320
1986-1990	M	5.504	5.536	5.725	5.655	5.766	5.977	6.143	6.283	6.352	6.378
	SD	1.504	1.382	1.293	1.423	1.416	1.253	1.179	1.123	1.055	1.147
	n	377	183	436	620	1079	857	704	1234	926	3079
1991-1995	M	5.729	5.357	5.721	5.500	5.830	6.042	6.210	6.348	6.455	6.462
	SD	1.526	1.569	1.379	1.480	1.322	1.200	1.146	1.107	0.996	1.057
	n	48	28	154	358	822	598	491	977	673	2506
Total	M	5.393	5.286	5.407	5.483	5.602	5.730	5.836	6.017	6.091	6.142
	SD	1.664	1.639	1.583	1.593	1.578	1.440	1.408	1.344	1.317	1.372
	N	6062	4125	6499	10079	17278	14973	13283	20686	16063	43473

Supplementary Table 5. Mean and standard deviation of “Climate change is caused by humans” for each cohort and survey wave

		Survey Wave (year)									
		1 (2009)	2 (2010)	3 (2011)	4 (2012)	5 (2013)	6 (2014)	7 (2015)	8 (2016)	9 (2017)	10 (2018)
1936-1940	M	4.671	4.380	4.303	4.411	4.581	4.783	4.842	5.038	5.162	5.068
	SD	1.851	1.931	1.887	1.797	1.839	1.706	1.697	1.676	1.626	1.753
	n	316	250	370	360	339	322	291	289	234	235
1941-1945	M	4.456	4.453	4.458	4.477	4.614	4.775	4.915	5.046	5.070	5.260
	SD	1.916	1.767	1.829	1.805	1.813	1.759	1.730	1.706	1.730	1.707
	n	419	340	552	652	637	609	576	582	501	580
1946-1950	M	4.536	4.468	4.455	4.538	4.701	4.902	4.943	5.192	5.229	5.249
	SD	1.801	1.774	1.827	1.769	1.790	1.713	1.766	1.659	1.657	1.763
	n	616	457	728	1044	1095	1060	990	1028	892	993
1951-1955	M	4.692	4.684	4.646	4.654	4.896	5.052	5.216	5.342	5.417	5.380
	SD	1.834	1.749	1.746	1.798	1.775	1.680	1.693	1.631	1.628	1.739
	n	650	490	758	1163	1657	1549	1411	2430	1955	5150
1956-1960	M	4.895	4.787	4.806	4.902	4.996	5.141	5.243	5.429	5.466	5.451
	SD	1.730	1.775	1.724	1.727	1.763	1.635	1.649	1.596	1.598	1.675
	n	722	531	788	1325	2635	2268	2096	3260	2606	7122
1961-1965	M	4.812	4.752	4.772	4.873	5.031	5.187	5.316	5.453	5.493	5.504
	SD	1.684	1.730	1.710	1.712	1.708	1.601	1.574	1.530	1.537	1.645
	n	735	521	772	1284	2477	2213	1960	3056	2344	6544
1966-1970	M	4.887	4.822	4.936	5.010	5.142	5.248	5.400	5.571	5.605	5.612
	SD	1.639	1.703	1.635	1.609	1.640	1.582	1.538	1.501	1.499	1.581
	n	736	478	641	1081	2161	1846	1646	2560	1979	5366
1971-1975	M	5.005	5.080	5.059	5.156	5.252	5.426	5.566	5.708	5.759	5.799
	SD	1.660	1.639	1.572	1.627	1.629	1.508	1.468	1.449	1.437	1.501
	n	580	376	523	890	1845	1561	1350	2181	1658	4685
1976-1980	M	5.157	5.136	5.190	5.279	5.407	5.467	5.643	5.802	5.902	5.890
	SD	1.567	1.590	1.510	1.541	1.558	1.534	1.483	1.393	1.356	1.444
	n	460	265	420	731	1430	1181	1014	1730	1284	3796
1981-1985	M	4.995	4.951	5.283	5.235	5.356	5.587	5.753	5.929	6.011	5.962
	SD	1.619	1.569	1.478	1.462	1.558	1.457	1.366	1.316	1.289	1.406
	n	364	184	315	515	1074	863	728	1322	965	3322
1986-1990	M	4.861	4.832	5.194	5.118	5.281	5.581	5.727	5.887	5.918	5.926
	SD	1.618	1.616	1.504	1.573	1.563	1.418	1.430	1.342	1.335	1.415
	n	375	184	434	620	1078	857	704	1234	924	3076
1991-1995	M	4.702	4.750	4.890	4.992	5.244	5.629	5.814	5.930	6.010	6.038
	SD	1.587	1.777	1.599	1.481	1.559	1.354	1.365	1.343	1.291	1.308
	n	47	28	154	357	823	596	494	977	671	2501
Total	M	4.816	4.745	4.795	4.882	5.082	5.238	5.363	5.556	5.598	5.644
	SD	1.729	1.739	1.716	1.700	1.696	1.605	1.598	1.531	1.530	1.593
	N	6020	4104	6455	10022	17251	14925	13260	20649	16013	43370

Examination of gender differences in climate change generation gap

This section of the Supplementary Material describes the results of additional analyses examining whether a generation gap in climate change beliefs would emerge when considering women and men separately. Supplementary Table 6 shows sample size and gender distribution was comparable for both climate change beliefs and across cohorts.

Results from the age-based trajectory model indicate that agreement with the reality of climate change was higher at all age groups than agreement with anthropogenic climate change for both women and men. This pattern of higher agreement levels for the reality of climate change than for anthropogenic climate change is evident when contrasting the darker lines from Supplementary Figures 1 to 4.

Agreement levels regarding both the reality of climate change and its human cause followed a U-shaped trajectory across the adult lifespan for both women and men (see Supplementary Figures 1-4). Results in Supplementary Table 7 confirm that the longitudinal changes in climate change beliefs did not follow a linear increase but a quadratic change trajectory for both climate change beliefs, as indicated by the statistically significant quadratic slopes (p-values $< .001$) for both women and men. There was one exception to this: women also showed a linear increase in the reality of climate change (p-value $= .001$). Overall, respondents' belief in the reality of climate change and belief in anthropogenic climate change was comparatively higher in early adulthood, lower in middle adulthood, at which point the pattern became higher in older ages; and this was equally observed for women and men.

Results in Supplementary Table 8 indicate that forcing a normative ageing effect to the data was less fitting than a cohort-based trajectory model allowing variability in each of the 12 five-year birth cohorts. Chi-square results in Supplementary Table 9 comparing the two models clearly indicate that the age-based trajectory model had relatively worse fit (reflected by higher chi-square values) for all birth cohorts compared to the cohort-based trajectory model.

We then examined the multi-group cohort sequential models estimating mean-level change over the ten annual assessments in each of the 12 five-year birth cohorts separately for women and men. Similar to findings for the overall sample, results indicate that agreement levels about the reality of climate change and human causation are increasing for people across all age cohorts at about the same rate, but older people are starting from a lower level of belief. As depicted in Supplementary Figure 1, all female age cohorts showed longitudinal patterns of increase for belief in the reality of climate change, except for the 1945-1941 cohort. For men, respondents' belief in the reality of climate change had longitudinal patterns of increase for just over half the age cohorts, except for the 1985-1981, 1960-1956, 1950-1946, 1945-1941 and 1940-1936 cohorts which shows longitudinal stability in agreement levels (see Supplementary Figure 2). Given the visual similarities in change trajectories, we also fitted a model to these data that formally tested whether each birth cohort's change trajectories were equal over time, while allowing their intercepts to vary. Results in Supplementary Table 8 indicate that this model was commensurate with the cohort-

unconstrained model, suggesting that beliefs in the reality of climate change had a similar growth rate for all birth cohorts over ten years amongst women and men.

Respondents' belief in the human causation of climate change had longitudinal patterns of increase for over half the female age cohorts, except for the 1990-1986, 1985-1981, 1950-1946 and 1945-1941 cohorts which shows longitudinal stability in agreement levels (see Supplementary Figure 3). As depicted in Supplementary Figure 4, most male age cohorts showed longitudinal patterns of increase for belief in the reality of climate change, except for the 1985-1981, 1950-1946 and 1940-1936 cohorts. Again, we also fitted a model that formally tested whether each birth cohort's change trajectories were equal over time, while allowing their intercepts to vary. Similar to findings for climate reality, results in Supplementary Table 8 suggest that beliefs in human causation had a similar growth rate for all birth cohorts over ten years amongst women and men. Supplementary Tables 10-13 provide more information about these models.

Supplementary Table 6. Age and sample sizes by birth cohort for "Climate change is real" (reality) and "Climate change is caused by humans" (cause) by gender. The youngest age in each birth cohort was taken as indication of age at Time 1.

			Sample Sizes			
Birth cohorts	Age at Time 1 (~2009)	Age at Time 10 (~2018)	Climate Reality		Climate Cause	
			Women	Men	Women	Men
1995-1991	18	27	2302	1082	2299	1080
1990-1986	19	28	2888	1399	2890	1395
1985-1981	24	33	2973	1416	2974	1414
1980-1976	29	38	3305	1752	3301	1751
1975-1971	34	43	3972	2116	3968	2115
1970-1966	39	48	4424	2570	4413	2564
1965-1961	44	53	5068	3136	5054	3137
1960-1956	49	58	5217	3594	5202	3589
1955-1951	54	63	3548	2713	3541	2701
1950-1946	59	68	867	697	867	696
1945-1941	64	73	494	442	494	439
1940-1936	69	78	245	250	246	249
Total n	—	—	35303	21167	35249	21130
Total N			56470		56379	

Supplementary Table 7. Parameter coefficients for the cohort-constrained models for "Climate change is real" (reality) and "Climate change is caused by humans" (cause) estimating the change trajectory from ages 18 to 78 by gender

		Climate Reality							
		Means				95% CIs		Variances	
		Estimate	SE	Est./SE	*p*-value	LB	UB	Est	SE
Women	Intercept	6.01	0.01	676.54	< .001	5.99	6.02	1.18**	0.01
	Linear slope	0.02	0.01	3.42	.001	0.01	0.03	0.06**	0.01
	Quadratic slope	0.03	0.00	8.08	< .001	0.02	0.04	0.00	0.00
Men	Intercept	5.73	0.01	495.83	< .001	5.71	5.76	1.18**	0.01
	Linear slope	0.00	0.01	0.10	.921	-0.01	0.01	0.06**	0.01
	Quadratic slope	0.04	0.00	9.94	< .001	0.04	0.05	0.00	0.00
		Climate Cause							
		Means				95% CIs		Variances	
		Estimate	SE	Est./SE	*p*-value	LB	UB	Est	SE
Women	Intercept	5.56	0.01	557.90	< .001	5.54	5.58	1.59**	0.02
	Linear slope	0.01	0.01	1.59	.112	-0.00	0.02	0.12**	0.01
	Quadratic slope	0.02	0.00	5.48	< .001	0.01	0.03	0.00	0.00
Men	Intercept	5.20	0.01	401.08	< .001	5.18	5.23	1.59**	0.02
	Linear slope	-0.02	0.01	-1.90	.058	-0.03	0.00	0.12**	0.01
	Quadratic slope	0.03	0.01	6.95	< .001	0.03	0.04	0.00	0.00

** $p < .001$

Supplementary Table 8. Fit statistics for the age-based and cohort-based trajectory models for each climate change belief for the multigroup gender models

Belief	Model	χ^2	*df*	*p*	$\Delta\chi^2$	*Δdf*	CFI	ΔCFI	RMSEA	ΔRMSEA	SRMR	ΔSRMR	AIC	Sample-size adjusted BIC
Climate Change Reality	Age-based trajectory (constrained)	22456.90	1550	< .001	—	—	0.725	—	0.076	—	0.227	—	480234.77	480292.40
	Cohort-based trajectory (constrained slopes)	14727.12	1528	< .001	7729.78	22	0.826	-0.101	0.061	0.015	0.175	0.052	472548.98	472733.41
	Cohort-based trajectory (unconstrained)	14432.62	1484	< .001	294.50	44	0.829	-0.003	0.061	0.000	0.178	-0.003	472342.48	472780.50
Climate Change Caused by Humans	Age-based trajectory (constrained)	19498.22	1550	< .001	—	—	0.789	—	0.070	—	0.179	—	500959.15	501016.77
	Cohort-based trajectory (constrained slopes)	10660.34	1528	< .001	8837.88	22	0.893	-0.104	0.050	0.020	0.130	0.049	492165.27	492349.65
	Cohort-based trajectory (unconstrained)	10425.22	1484	< .001	235.12	44	0.895	-0.002	0.051	-0.001	0.131	-0.001	492018.16	492456.05

χ^2 = chi-square. *df* = degrees of freedom. *p* = *p*-value. CFI = Comparative Fit Index. RMSEA = Root Mean Square Error of Approximation. SRMR = Standardized Root Mean Square Residual. AIC = Akaike Information Criterion. BIC = Bayesian Information Criterion.

Supplementary Table 9. Chi-square (χ^2) contributions and differences of each birth cohort for "Climate change is real" (reality) and "Climate change is caused by humans" (cause) by gender

	Climate Reality									
	Women					Men				
Birth cohort	χ^2A	χ^2B	$\chi^2\Delta$AB	χ^2C	$\chi^2\Delta$BC	χ^2A	χ^2B	$\chi^2\Delta$AB	χ^2C	$\chi^2\Delta$BC
1995-1991	1363	909	455	875	34	620	391	229	377	14
1990-1986	1468	883	585	852	31	799	516	284	507	9
1985-1981	1563	1006	557	992	14	567	381	186	378	3
1980-1976	1403	961	442	925	35	593	368	225	365	3
1975-1971	1352	871	482	841	29	649	449	200	447	2
1970-1966	1282	810	472	781	29	737	531	206	528	3
1965-1961	1249	750	499	738	12	982	678	303	677	2
1960-1956	1147	725	423	694	30	928	655	274	655	0
1955-1951	1120	667	453	661	6	652	506	146	503	3
1950-1946	907	510	397	496	15	816	564	252	565	-1
1945-1941	524	289	235	285	5	727	518	209	514	4
1940-1936	471	346	125	332	14	537	444	92	445	0

	Climate Cause									
Birth cohort	Women					Men				
	χ^2A	χ^2B	$\chi^2\Delta$AB	χ^2C	$\chi^2\Delta$BC	χ^2A	χ^2B	$\chi^2\Delta$AB	χ^2C	$\chi^2\Delta$BC
1995-1991	997	475	522	437	38	540	299	242	268	30
1990-1986	1261	604	657	591	13	743	403	340	379	24
1985-1981	1207	629	579	627	1	481	277	204	277	1
1980-1976	1185	637	548	622	15	537	334	203	327	7
1975-1971	1141	570	571	557	13	748	498	251	494	4
1970-1966	1119	575	543	570	5	723	474	249	471	3
1965-1961	1091	517	575	513	4	789	511	277	507	4
1960-1956	983	453	531	439	13	811	493	318	493	0
1955-1951	1030	446	584	441	6	700	492	208	490	2
1950-1946	925	440	485	426	14	617	353	264	349	4
1945-1941	567	291	276	287	4	473	281	192	267	14
1940-1936	493	395	98	387	9	336	213	123	205	9

Chi-square (χ^2) contribution differences ($\chi^2\Delta$) between the key competing models are shown in bold. χ^2A = Chi-square contribution of the age-based (constrained) model. χ^2B = Chi-square contribution of the intermediate model where intercepts are freely estimated but slopes are constrained. χ^2C= Chi-square contribution of the cohort-based (unconstrained) model.

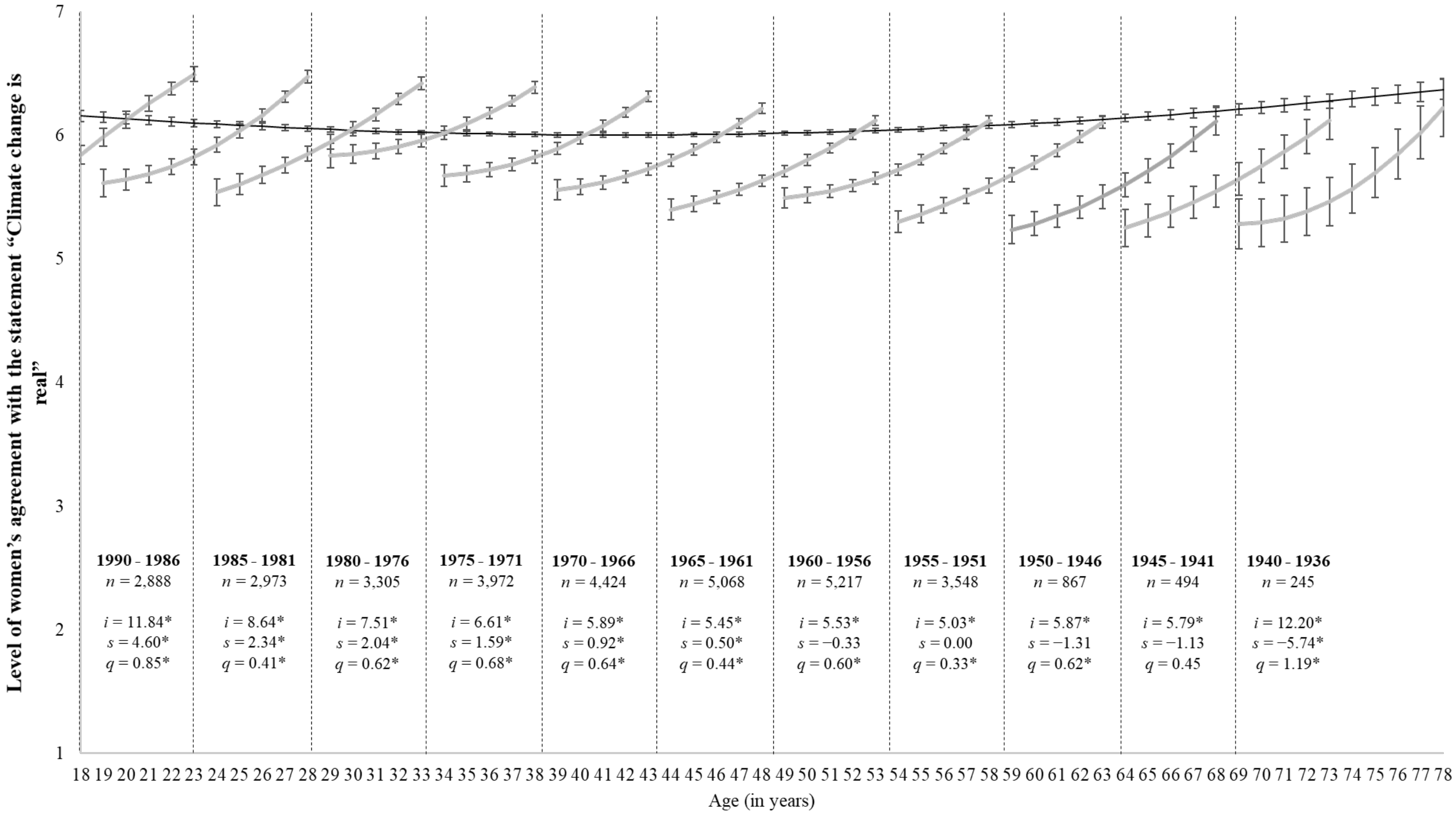

Supplementary Figure 1. Belief in the reality of climate change across age and five-year birth cohorts among women. Model-implied change trajectories in the level of women's agreement with the statement "Climate change is real" (dark line) from ages 18 to 78. The lines within each 5-year cohort represent longitudinal change in climate reality estimating latent intercepts (i) as well as linear (s) and quadratic (q) slopes. The estimations are based on the mean-levels of climate reality (y-axis) across age and assessments (x-axis) with 95% confidence intervals presented as error bars around each point estimate (* $p < .05$). Due to graphical space constraints, details for the 1995-1991 cohort are not shown but are: $n = 2302$, $i = 7.15$*, $s = -0.53$, $q = -0.38$. Respondents expressed their levels of disagreement-agreement on a 7-point answer scale anchored by 1 (strongly disagree) and 7 (strongly agree).

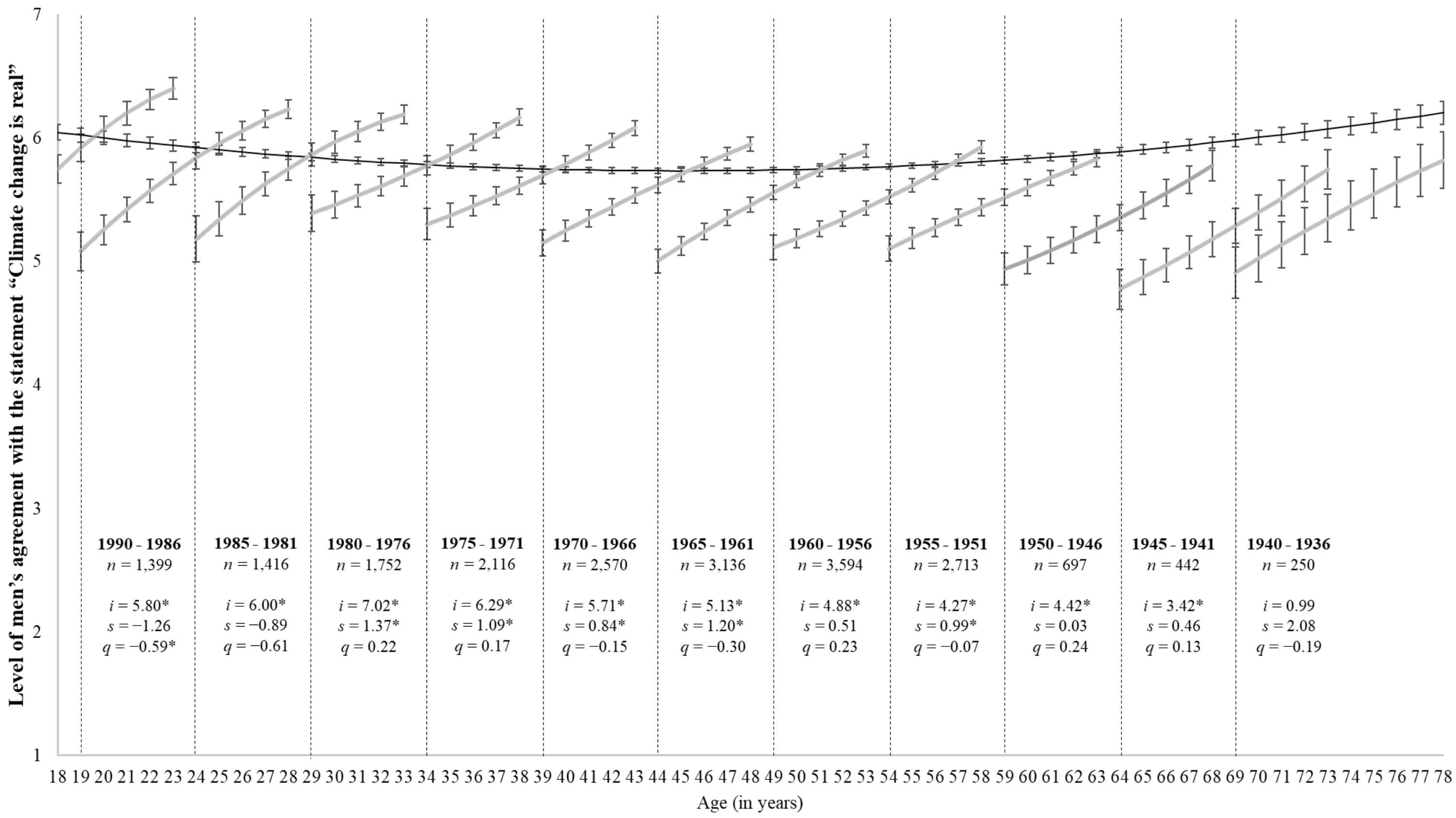

Supplementary Figure 2. Belief in the reality of climate change across age and five-year birth cohorts among men. Model-implied change trajectories in the level of men's agreement with the statement "Climate change is real" (dark line) from ages 18 to 78. The lines within each 5-year cohort represent longitudinal change in climate reality estimating latent intercepts (*i*) as well as linear (*s*) and quadratic (*q*) slopes. The estimations are based on the mean-levels of climate reality (y-axis) across age and assessments (x-axis) with 95% confidence intervals presented as error bars around each point estimate (* $p < .05$). Due to graphical space constraints, details for the 1995-1991 cohort are not shown but are: $n = 1082$, $i = 3.30$, $s = -3.62$, $q = -1.01$*. Respondents expressed their levels of disagreement-agreement on a 7-point answer scale anchored by 1 (strongly disagree) and 7 (strongly agree).

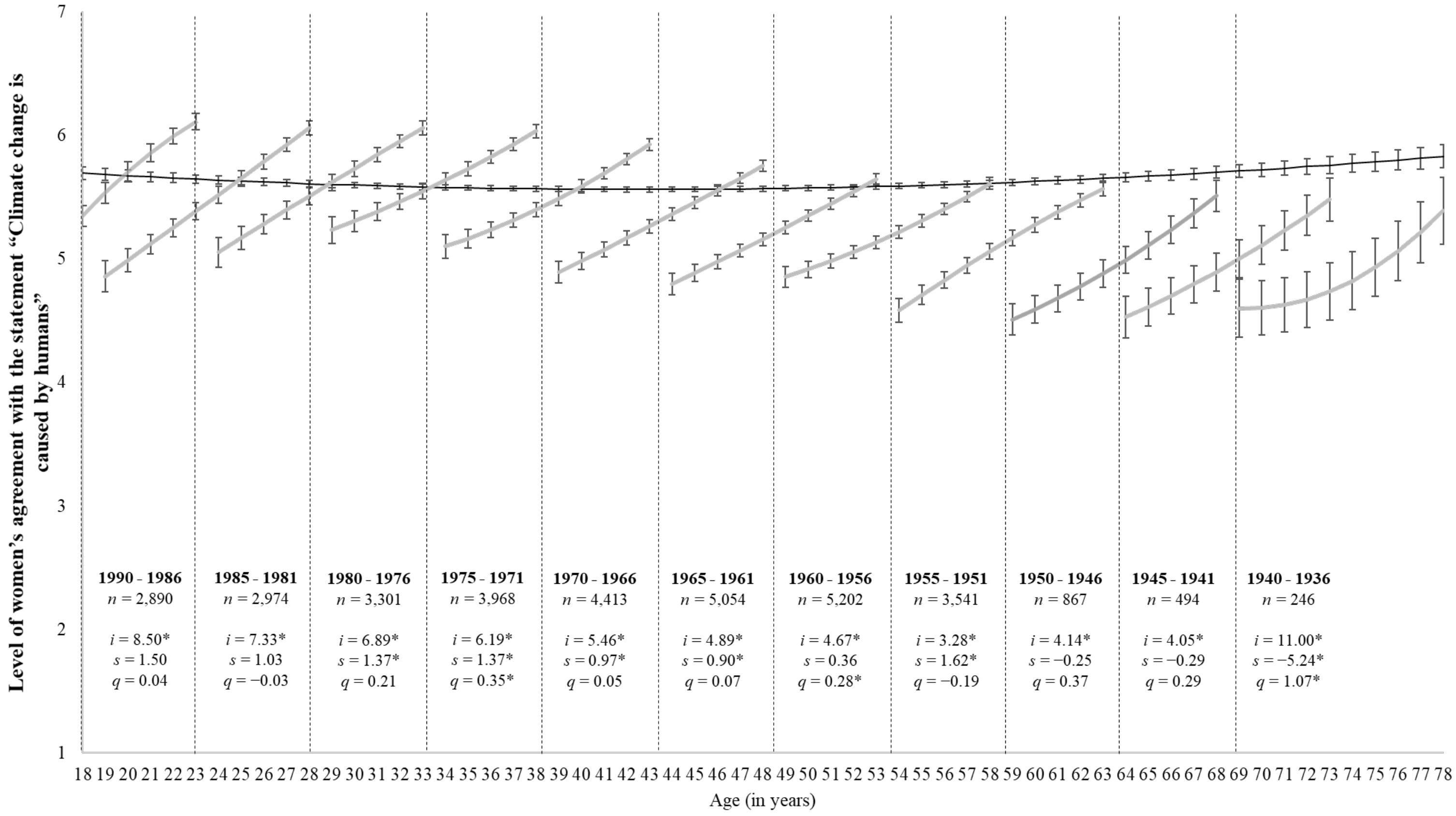

Supplementary Figure 3. Belief in anthropogenic climate change across age and five-year birth cohorts among women. Model-implied change trajectories in the level of women's agreement with the statement "Climate change is caused by humans" (dark line) from ages 18 to 78. The lines within each 5-year cohort represent longitudinal change in climate cause belief estimating latent intercepts (*i*) as well as linear (*s*) and quadratic (*q*) slopes. The estimations are based on the mean-levels of climate cause belief (y-axis) across age and assessments (x-axis) with 95% confidence intervals presented as error bars around each point estimate (* $p < .05$). Due to graphical space constraints, details for the 1995-1991 cohort are not shown but are: n = 2299, i = 4.20, s = -2.82, q = -0.89*. Respondents expressed their levels of disagreement-agreement on a 7-point answer scale anchored by 1 (strongly disagree) and 7 (strongly agree).

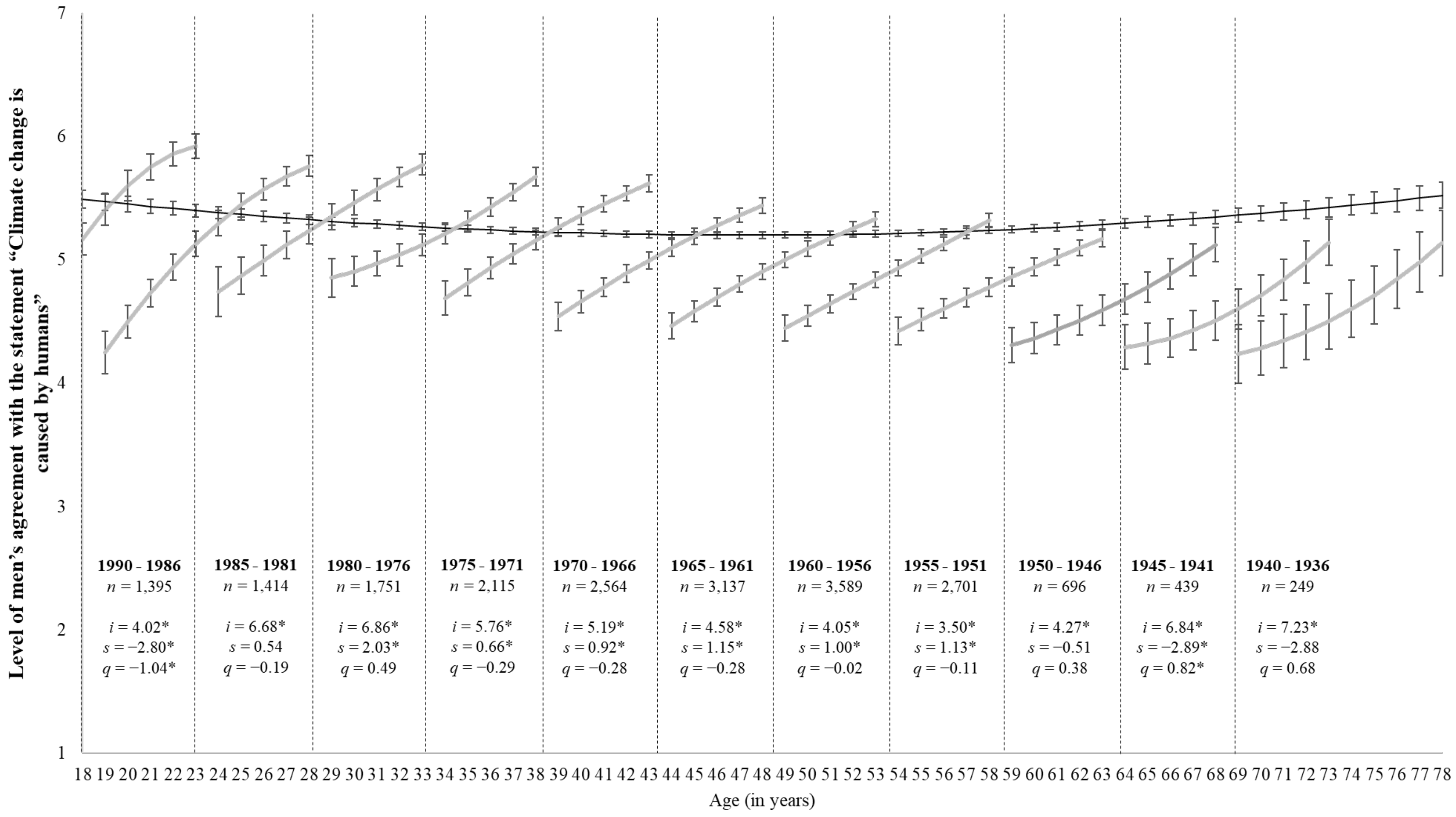

Supplementary Figure 4. Belief in anthropogenic climate change across age and five-year birth cohorts among men. Model-implied change trajectories in the level of men's agreement with the statement "Climate change is caused by humans" (dark line) from ages 18 to 78. The lines within each 5-year cohort represent longitudinal change in climate cause belief estimating latent intercepts (*i*) as well as linear (*s*) and quadratic (*q*) slopes. The estimations are based on the mean-levels of climate cause belief (y-axis) across age and assessments (x-axis) with 95% confidence intervals presented as error bars around each point estimate (* $p < .05$). Due to graphical space constraints, details for the 1995-1991 cohort are not shown but are: $n = 1080$, $i = -4.06$, $s = -9.46$*, $q = -2.24$*. Respondents expressed their levels of disagreement-agreement on a 7-point answer scale anchored by 1 (strongly disagree) and 7 (strongly agree).

Supplementary Table 10. Estimates for the cohort-based unconstrained models for “Climate change is real” (reality) for women and men

		Climate Reality											
		Women						Men					
						95% CI						95% CI	
Birth cohort		Est.	SE	Est./SE	*p*-value	LB	UB	Est.	SE	Est./SE	*p*-value	LB	UB
1995-1991	*i*	7.15	2.04	3.51	< .001	3.16	11.14	3.30	3.14	1.05	.293	-2.85	9.45
	s	-0.53	1.64	-0.32	.747	-3.74	2.68	-3.62	2.53	-1.43	.152	-8.58	1.33
	q	-0.38	0.33	-1.15	.251	-1.02	0.27	-1.01	0.50	-1.99	.046	-1.99	-0.02
1990-1986	*i*	11.84	0.84	14.11	< .001	10.20	13.49	5.80	1.19	4.86	< .001	3.46	8.14
	s	4.60	0.83	5.56	< .001	2.98	6.21	-1.26	1.17	-1.08	.282	-3.55	1.04
	q	0.85	0.20	4.24	< .001	0.46	1.24	-0.59	0.28	-2.10	.036	-1.14	-0.04
1985-1981	*i*	8.64	0.45	19.13	< .001	7.75	9.52	6.00	0.74	8.13	< .001	4.56	7.45
	s	2.34	0.59	3.94	< .001	1.18	3.50	-0.89	0.98	-0.92	.360	-2.81	1.02
	q	0.41	0.19	2.18	.029	0.04	0.78	-0.61	0.31	-1.95	.051	-1.22	0.00
1980-1976	*i*	7.51	0.18	41.12	< .001	7.15	7.87	7.02	0.27	26.38	< .001	6.50	7.55
	s	2.04	0.36	5.61	< .001	1.32	2.75	1.37	0.53	2.59	.010	0.33	2.41
	q	0.62	0.17	3.67	< .001	0.29	0.95	0.22	0.25	0.90	.369	-0.26	0.71
1975-1971	*i*	6.61	0.04	153.38	< .001	6.52	6.69	6.29	0.06	103.23	< .001	6.17	6.41
	s	1.59	0.17	9.17	< .001	1.25	1.94	1.09	0.25	4.38	< .001	0.60	1.58
	q	0.68	0.15	4.57	< .001	0.39	0.97	0.17	0.21	0.81	.419	-0.25	0.59
1970-1966	*i*	5.89	0.02	259.38	< .001	5.84	5.93	5.71	0.03	187.45	< .001	5.65	5.77
	s	0.92	0.04	20.84	< .001	0.83	1.01	0.84	0.06	13.96	< .001	0.73	0.96
	q	0.64	0.14	4.62	< .001	0.37	0.91	-0.15	0.18	-0.80	.425	-0.50	0.21
1965-1961	*i*	5.45	0.03	170.14	< .001	5.38	5.51	5.13	0.04	130.84	< .001	5.05	5.20
	s	0.50	0.12	4.04	< .001	0.26	0.74	1.20	0.15	8.12	< .001	0.91	1.50
	q	0.44	0.13	3.26	.001	0.17	0.70	-0.30	0.17	-1.81	.071	-0.62	0.03
1960-1956	*i*	5.53	0.11	49.50	< .001	5.31	5.75	4.88	0.14	35.04	< .001	4.60	5.15
	s	-0.33	0.25	-1.34	.179	-0.82	0.15	0.51	0.31	1.66	.098	-0.09	1.11
	q	0.60	0.13	4.61	< .001	0.35	0.86	0.23	0.16	1.43	.153	-0.09	0.55
1955-1951	*i*	5.03	0.29	17.10	< .001	4.46	5.61	4.27	0.35	12.33	< .001	3.59	4.95
	s	0.00	0.43	0.01	.995	-0.83	0.84	0.99	0.50	1.97	.049	0.01	1.98
	q	0.33	0.15	2.21	.027	0.04	0.62	-0.07	0.18	-0.39	.695	-0.42	0.28
1950-1946	*i*	5.87	0.66	8.95	< .001	4.58	7.15	4.42	0.73	6.05	< .001	2.99	5.85
	s	-1.31	0.71	-1.86	.063	-2.70	0.07	0.03	0.79	0.04	.968	-1.51	1.57
	q	0.62	0.19	3.31	.001	0.25	0.99	0.24	0.21	1.17	.243	-0.17	0.65
1945-1941	*i*	5.79	1.35	4.28	< .001	3.14	8.44	3.42	1.47	2.33	.020	0.55	6.30
	s	-1.13	1.15	-0.98	.326	-3.39	1.13	0.46	1.25	0.37	.711	-1.98	2.90
	q	0.45	0.24	1.84	.065	-0.03	0.92	0.13	0.26	0.50	.614	-0.38	0.64
1940-1936	*i*	12.20	2.64	4.61	< .001	7.02	17.38	0.99	2.73	0.36	.716	-4.35	6.34
	s	-5.74	1.87	-3.06	.002	-9.41	-2.07	2.08	1.92	1.08	.279	-1.69	5.85
	q	1.19	0.33	3.62	< .001	0.55	1.84	-0.19	0.34	-0.56	.578	-0.85	0.47

i = intercept, *s* = linear slope, *q* = quadratic slope.

Supplementary Table 11. Estimates for the cohort-based unconstrained models for “Climate change is caused by humans” (cause) for women and men

		Climate Cause											
		Women						Men					
						95% CI						95% CI	
Birth cohort		Est	SE	Est./SE	*p*-value	LB	UB	Est	SE	Est./SE	*p*-value	LB	UB
1995-1991	*i*	4.20	2.16	1.94	.052	-0.04	8.44	-4.06	3.38	-1.20	.229	-10.69	2.56
	s	-2.82	1.74	-1.62	.105	-6.23	0.59	-9.46	2.72	-3.48	.001	-14.80	-4.13
	q	-0.89	0.35	-2.56	.010	-1.57	-0.21	-2.24	0.54	-4.12	< .001	-3.30	-1.17
1990-1986	*i*	8.50	0.89	9.51	< .001	6.75	10.25	4.02	1.27	3.16	.002	1.52	6.51
	s	1.50	0.88	1.71	.088	-0.22	3.22	-2.80	1.25	-2.24	.025	-5.24	-0.35
	q	0.04	0.21	0.18	.855	-0.38	0.46	-1.04	0.30	-3.48	.001	-1.63	-0.45
1985-1981	*i*	7.33	0.48	15.35	< .001	6.40	8.27	6.68	0.79	8.48	< .001	5.14	8.23
	s	1.03	0.63	1.64	.102	-0.20	2.26	0.54	1.04	0.51	.608	-1.51	2.58
	q	-0.03	0.20	-0.14	.888	-0.42	0.36	-0.19	0.33	-0.55	.580	-0.84	0.47
1980-1976	*i*	6.89	0.19	35.72	< .001	6.51	7.27	6.86	0.28	24.32	< .001	6.31	7.42
	s	1.37	0.38	3.59	< .001	0.62	2.13	2.03	0.56	3.61	< .001	0.93	3.13
	q	0.21	0.18	1.19	.233	-0.14	0.56	0.49	0.26	1.85	.064	-0.03	1.00
1975-1971	*i*	6.19	0.05	134.65	< .001	6.10	6.28	5.76	0.07	88.62	< .001	5.63	5.89
	s	1.37	0.18	7.51	< .001	1.02	1.73	0.66	0.26	2.50	.012	0.14	1.17
	q	0.35	0.16	2.26	.024	0.05	0.66	-0.29	0.23	-1.29	.196	-0.73	0.15
1970-1966	*i*	5.46	0.03	216.10	< .001	5.41	5.51	5.19	0.03	153.54	< .001	5.12	5.26
	s	0.97	0.05	20.76	< .001	0.88	1.06	0.92	0.06	14.31	< .001	0.79	1.04
	q	0.05	0.14	0.32	.748	-0.24	0.33	-0.28	0.19	-1.45	.146	-0.65	0.10
1965-1961	*i*	4.89	0.04	141.02	< .001	4.82	4.95	4.58	0.04	107.99	< .001	4.50	4.67
	s	0.90	0.13	6.94	< .001	0.64	1.15	1.15	0.16	7.43	< .001	0.85	1.46
	q	0.07	0.14	0.49	.623	-0.21	0.34	-0.28	0.17	-1.60	.110	-0.61	0.06
1960-1956	*i*	4.67	0.12	39.85	< .001	4.44	4.89	4.05	0.15	27.64	< .001	3.76	4.34
	s	0.36	0.26	1.38	.166	-0.15	0.87	1.00	0.32	3.09	.002	0.37	1.63
	q	0.28	0.14	2.07	.039	0.02	0.55	-0.02	0.17	-0.10	.921	-0.35	0.32
1955-1951	*i*	3.28	0.31	10.66	< .001	2.68	3.89	3.50	0.36	9.65	< .001	2.79	4.21
	s	1.62	0.45	3.62	< .001	0.74	2.49	1.13	0.53	2.15	.032	0.10	2.16
	q	-0.19	0.16	-1.23	.218	-0.50	0.11	-0.11	0.19	-0.60	.547	-0.47	0.25
1950-1946	*i*	4.14	0.68	6.07	< .001	2.80	5.48	4.27	0.76	5.63	< .001	2.79	5.76
	s	-0.25	0.73	-0.35	.730	-1.69	1.18	-0.51	0.82	-0.63	.529	-2.11	1.09
	q	0.37	0.19	1.90	.057	-0.01	0.75	0.38	0.22	1.78	.076	-0.04	0.81
1945-1941	*i*	4.05	1.41	2.88	.004	1.29	6.81	6.84	1.52	4.49	< .001	3.86	9.83
	s	-0.29	1.20	-0.24	.808	-2.64	2.06	-2.89	1.29	-2.24	.025	-5.42	-0.36
	q	0.29	0.25	1.14	.256	-0.21	0.78	0.82	0.27	3.01	.003	0.29	1.35
1940-1936	*i*	11.00	2.76	3.98	< .001	5.58	16.41	7.23	2.82	2.56	.010	1.70	12.76
	s	-5.24	1.96	-2.68	.007	-9.08	-1.41	-2.88	1.99	-1.45	.148	-6.78	1.02
	q	1.07	0.34	3.12	.002	0.40	1.75	0.68	0.35	1.96	.051	-0.00	1.36

i = intercept, *s* = linear slope, *q* = quadratic slope.

Supplementary Table 12. Parameter coefficients for the partially-constrained cohort-based models for "Climate change is real" (reality) for women and men

Climate Reality									
		Means				95% CIs		Variances	
Women		Estimate	SE	Est./SE	*p*-value	LB	UB	Est	SE
1995-1991		8.45	0.05	158.62	< .001	8.35	8.56	1.13*	0.01
1990-1986		7.96	0.04	191.91	< .001	7.88	8.04	1.13*	0.01
1985-1981		7.48	0.03	231.81	< .001	7.41	7.54	1.13*	0.01
1980-1976		7.04	0.03	277.36	< .001	6.99	7.09	1.13*	0.01
1975-1971	Intercepts	6.49	0.02	316.77	< .001	6.45	6.53	1.13*	0.01
1970-1966	freely	5.94	0.02	311.86	< .001	5.90	5.97	1.13*	0.01
1965-1961	estimated	5.38	0.02	266.64	< .001	5.35	5.42	1.13*	0.01
1960-1956		4.94	0.03	199.76	< .001	4.89	4.99	1.13*	0.01
1955-1951		4.46	0.03	130.06	< .001	4.39	4.53	1.13*	0.01
1950-1946		3.93	0.06	71.94	< .001	3.82	4.04	1.13*	0.01
1945-1941		3.51	0.08	46.69	< .001	3.37	3.66	1.13*	0.01
1940-1936		3.04	0.11	28.33	< .001	2.83	3.25	1.13*	0.01
All cohorts	Linear slope constrained	0.91	0.01	64.46	< .001	0.88	0.94	0.05**	0.01
All cohorts	Quadratic slope constrained	-0.00	0.01	-0.71	.479	-0.01	0.01	0.00	0.00

		Means				95% CIs		Variances	
Men		Estimate	SE	Est./SE	*p*-value	LB	UB	Est	SE
1995-1991		8.56	0.08	110.75	< .001	8.41	8.71	1.13*	0.01
1990-1986		7.90	0.06	131.51	< .001	7.79	8.02	1.13*	0.01
1985-1981		7.39	0.05	158.85	< .001	7.30	7.48	1.13*	0.01
1980-1976		6.86	0.04	193.28	< .001	6.79	6.93	1.13*	0.01
1975-1971	Intercepts	6.29	0.03	222.67	< .001	6.23	6.34	1.13*	0.01
1970-1966	freely	5.69	0.03	226.92	< .001	5.64	5.74	1.13*	0.01
1965-1961	estimated	5.15	0.03	200.06	< .001	5.10	5.20	1.13*	0.01
1960-1956		4.69	0.03	154.89	< .001	4.63	4.75	1.13*	0.01
1955-1951		4.17	0.04	103.00	< .001	4.09	4.25	1.13*	0.01
1950-1946		3.60	0.06	58.09	< .001	3.48	3.72	1.13*	0.01
1945-1941		3.05	0.08	37.20	< .001	2.89	3.21	1.13*	0.01
1940-1936		2.74	0.11	24.80	< .001	2.53	2.96	1.13*	0.01
All cohorts	Linear slope constrained	0.96	0.02	50.45	<.001	0.93	1.00	0.05*	0.01
All cohorts	Quadratic slope constrained	-0.02	0.01	-2.24	.025	-0.03	-0.00	0.00	0.00

$* p < .001$

Supplementary Table 13. Parameter coefficients for the partially-constrained cohort-based models for "Climate change is caused by humans" (cause) for women and men

Climate Cause									
		Means				95% CIs		Variances	
Women		Estimate	SE	Est./SE	*p*-value	LB	UB	Est	SE
1995-1991		8.46	0.06	145.26	< .001	8.35	8.58	1.54*	0.02
1990-1986		7.85	0.05	173.14	< .001	7.76	7.94	1.54*	0.02
1985-1981		7.34	0.04	206.14	< .001	7.27	7.41	1.54*	0.02
1980-1976		6.80	0.03	239.62	< .001	6.74	6.85	1.54*	0.02
1975-1971	Intercepts freely estimated	6.14	0.02	265.58	< .001	6.09	6.18	1.54*	0.02
1970-1966		5.46	0.02	252.92	< .001	5.41	5.50	1.54*	0.02
1965-1961		4.84	0.02	213.53	< .001	4.79	4.88	1.54*	0.02
1960-1956		4.31	0.03	157.75	< .001	4.26	4.36	1.54*	0.02
1955-1951		3.76	0.04	100.01	< .001	3.69	3.84	1.54*	0.02
1950-1946		3.11	0.06	50.65	< .001	2.99	3.23	1.54*	0.02
1945-1941		2.64	0.09	31.07	< .001	2.48	2.81	1.54*	0.02
1940-1936		2.12	0.12	17.32	< .001	1.88	2.36	1.54*	0.02
All cohorts	Linear slope constrained	1.04	0.02	69.31	< .001	1.01	1.07	0.10**	0.01
All cohorts	Quadratic slope constrained	-0.02	0.01	-4.26	< .001	-0.03	-0.01	0.00	0.00

		Means				95% CIs		Variances	
Men		Estimate	SE	Est./SE	*p*-value	LB	UB	Est	SE
1995-1991		8.40	0.09	99.15	< .001	8.23	8.56	1.54*	0.02
1990-1986		7.63	0.07	115.83	< .001	7.50	7.76	1.54*	0.02
1985-1981		7.10	0.05	137.81	< .001	7.00	7.20	1.54*	0.02
1980-1976		6.43	0.04	162.37	< .001	6.35	6.51	1.54*	0.02
1975-1971	Intercepts freely estimated	5.86	0.03	184.10	< .001	5.80	5.92	1.54*	0.02
1970-1966		5.16	0.03	181.76	< .001	5.11	5.22	1.54*	0.02
1965-1961		4.56	0.03	157.78	< .001	4.50	4.61	1.54*	0.02
1960-1956		4.04	0.03	120.94	< .001	3.98	4.11	1.54*	0.02
1955-1951		3.47	0.04	78.20	< .001	3.39	3.56	1.54*	0.02
1950-1946		2.90	0.07	41.70	< .001	2.76	3.04	1.54*	0.02
1945-1941		2.41	0.09	26.07	< .001	2.23	2.59	1.54*	0.02
1940-1936		1.97	0.13	15.66	< .001	1.72	2.21	1.54*	0.02
All cohorts	Linear slope constrained	1.04	0.02	50.84	<.001	1.00	1.08	0.10*	0.01
All cohorts	Quadratic slope constrained	-0.05	0.01	-6.24	< .001	-0.06	-0.03	0.00	0.00

* $p < .001$
